# A Barrage Jamming Strategy Based on CRB Maximization against Distributed MIMO Radar

**DOI:** 10.3390/s19112453

**Published:** 2019-05-29

**Authors:** Guangyong Zheng, Siqi Na, Tianyao Huang, Lulu Wang

**Affiliations:** 1 State Key Laboratory of Complex Electromagnetic Environment Effects on Electronic Information Systems (CEMEE), Luoyang 471003, China; lightgg@ustc.edu; 2 Department of Electronic Engineering, Tsinghua University, Beijing 100084, China; nasiqi123456@163.com; 3 Artificial Intelligence Research Center, National Innovation Institute of Defense Technology, Academy of Military Science, Beijing 100071, China; wanglulunudt@163.com

**Keywords:** Barrage jamming, Distributed MIMO radar, Cramer–Rao bound

## Abstract

Distributed multiple input multiple output (MIMO) radar has attracted much attention for its improved detection and estimation performance as well as enhanced electronic counter-counter measures (ECCM) ability. To protect the target from being detected and tracked by such radar, we consider a barrage jamming strategy towards a distributed MIMO. We first derive the Cramer–Rao bound (CRB) of target parameters estimation using a distributed MIMO under barrage jamming environments. We then set maximizing the CRB as the criterion for jamming resource allocation, aiming at degrading the accuracy of target parameters estimation. Due to the non-convexity of the CRB maximizing problem, particle swarm optimization is used to solve the problem. Simulation results demonstrate the advantages of the proposed strategy over traditional jamming methods.

## 1. Introduction

The idea of multiple input multiple output (MIMO) radar, which uses multiple antennas at transmit and receive sides, has been widely studied by academic and industrial researchers [1,2]. MIMO radars are generally divided into two categories: collocated [1] and distributed [2], which refer to using closely placed and widely separated radar antennas, respectively. In this paper, we focus on distributed MIMO radar for its merits of spatial diversity. Due to the spatial diversity, higher resolution of target location and better target estimation performance is achieved. In particular, spatial diversity significantly enhances the electronic counter-counter measures (ECCM) ability of the radar, which becomes a threat of current electronic warfare devices.

Along with the development of distributed MIMO radar, designing the jamming strategies against distributed MIMO radar becomes an important topic. In Ref. [3], the authors propose a jamming criterion that minimizes the mutual information of the radar, and use the criterion to optimize the jamming power allocation. However, the echo of the distributed radar is modeled as a linear combination of the transmitted waveform in Ref. [3], and the delay and Doppler effect are not taken into account, which restricts the application of the strategy to static target scenarios. Similar criteria are further discussed in Refs. [4,5,6], and game theory is introduced in Refs. [7,8] to analyze the power allocation of radar and jammer. However, the signal models are inherited from Ref. [3], and required to be broadened to moving target scenarios. Many barrage jamming techniques have been developed accounting Doppler effects of radar targets [9,10,11,12,13,14]. In Refs. [9,10,11,12,13], several barrage jamming strategies are proposed for mono-static radar, and the jamming performance on radar imaging or target detection is evaluated. However, these approaches are proposed for mono-static radar, and are not directly applicable for distributed antenna setups. Shen et al. in Ref. [14] evaluate the jamming effect against a radar network consisting of multiple mono-static radars, while these radars do not perform joint signal processing as in a distributed MIMO radar.

In this paper, we consider a moving target scenario and develop a barrage jamming strategy towards the MIMO radar for protecting the target from being detected or tracked. The goal of the jamming device is to minimize the radar’s accuracy of target parameters (e.g., location or velocity) by optimizing the barrage jamming power allocated for radar antennas. In contrast to the mutual information used in Ref. [3], we use Cramer–Rao bound (CRB), as an approximation of the estimation error. Since CRB is the lower bound of the root mean square error of the unbiased estimator, maximizing CRB possibly leads to increased radar estimation errors of target parameters, hence achieves the goal for protecting the target. The CRB-based jamming criterion is also expected to compatible with different signal processing methods of MIMO radars, because CRB is regardless of the signal processing methods.

To implement the barrage jamming towards distributed MIMO radar, we derive the CRB of the target location and motion parameters, as a function of jamming power allocation. We then construct the optimization problem that maximizes the CRB subject to a fixed budget of total jamming power. The resultant CRB maximization problem is highly non-convex, and we apply particle swarm optimization to obtain a numerical solution.

The remainder of the paper is organized as follows. Section 2 reviews the signal model of a distributed MIMO. CRB of the estimation of target parameters under jamming environment is present in Section 3. In Section 4, we construct the optimization model of the interference power allocation. Numerical results, which illustrate the performance of the proposed method, are shown in Section 5. Conclusions are drawn in Section 6.

## 2. Signal Model of MIMO Radar

**Figure 1:**
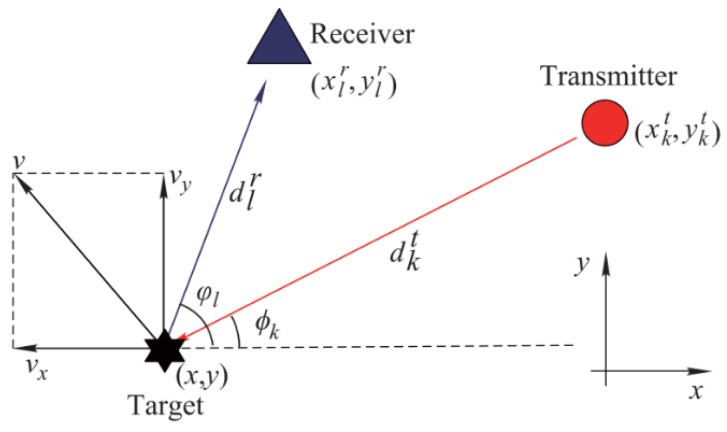
The geometry the radar and the target [15].

Assume that the target and all radar receive and transmit antennas are in a two-dimensional plane (which can be extended to three dimensions), as in Ref. [15]. The geometry of the radar and target is shown in Figure 1. Suppose that there are *K* transmit antennas and *L* receive antennas in the distributed MIMO radar. Denote by $\left(x_{k}^{t},y_{k}^{t}\right)$ and $\left(x_{l}^{r},y_{l}^{r}\right)$ the positions of the *k*th transmitting and the *l*th receiving unit, respectively, k=1,2,…,K, l=1,2,…,L. The target is located at (x,y), with the motion velocity $\left(v_{x},v_{y}\right)$. The distance from the *k*th transmit antenna to the target is expressed as

(1) $$d_{k}^{t}=\sqrt{{\left(x_{k}^{t}-x\right)}^{2}+{\left(y_{k}^{t}-y\right)}^{2}},$$

and the distance from the *l*th receive antenna to the target is

(2) $$d_{l}^{r}=\sqrt{{\left(x_{l}^{r}-x\right)}^{2}+{\left(y_{l}^{r}-y\right)}^{2}}.$$

Orthogonal baseband waveform vector, $s\left(t\right)={\left[s_{1}\left(t\right),\cdots ,s_{K}\left(t\right)\right]}^{T}$, is used by the MIMO radar, with the *k*th entry $s_{k}\left(t\right)$ satisfying the following condition

(3) $$\int_{T}s_{k}\left(t\right)s_{m}^{*}\left(t\right)dt\approx \left\{\begin{array}{cc} 1, & k=m,\\ 0, & k\neq m, \end{array}\right.$$

where T represents the duration of the radar baseband waveform and ${\left(\cdot \right)}^{*}$ denotes conjugate. In particular, we consider a set of narrowband, frequency orthogonal pulses, expressed as

(4) $$s_{k}\left(t\right)=\left\{\begin{array}{c} e^{j2\pi k\Delta ft}/\sqrt{T},0\leq t\leq T,\\ 0,else, \end{array}\right.$$

where Δf denotes the frequency step.

After up-conversion with $e^{j2pif_{c}t}$, where $f_{c}$ is the carrier frequency, these baseband signals are sent and scattered by the target. Receive antennas then receives the radar echoes, those transmitted signals that are reflected to the radar by the target. We define a vector $r\left(t\right)={\left[r_{1}\left(t\right),\cdots ,r_{L}\left(t\right)\right]}^{T}$, of which the *l*th element represents the radar echo received by the *l*th receive antenna and can be expressed as a superimposition of all transmissions (with delay and Doppler shift) from *K* transmit antennas [15,16], i.e.,

(5) $$r_{l}\left(t\right)=\sum_{k=1}^{K}\xi_{lk}s_{k}\left(t-\tau_{lk}\right)e^{-j2\pi f_{c}\tau_{lk}}e^{j2\pi f_{lk}t}+w_{l}\left(t\right),$$

where $\xi_{lk}$ denotes the received target scattering intensity, $\tau_{lk}$ denotes the delays of transmitter-to-target and target-to-receiver channels, i.e.,

(6) $$\tau_{lk}=\left(d_{k}^{t}+d_{l}^{r}\right)/c,$$

with *c* the speed of light. Doppler frequency shift $f_{lk}$ with respect to the *k*th transmit channel and the *l*th receive channel [17,18] is given by

(7) $$f_{lk}=\frac{v_{x}\left(f_{c}+k\Delta f\right)}{c}\left(\cos\phi_{k}+\cos\varphi_{l}\right)+\frac{v_{y}\left(f_{c}+k\Delta f\right)}{c}\left(\sin\phi_{k}+\sin\varphi_{l}\right),$$

where $\phi_{k}$ and $\phi_{l}$ denote the angles of the line of sights, i.e., the *k*th transmit antenna to the target and the *l*th receive antenna to the target, with respect to the *x* axis, respectively. The term $w_{l}\left(t\right)$ represents the sum of the receiver thermal noise and the interference towards the *l*th receiving channel. Both of the additive noises and barrage jamming signals are independently, identically distributed Gaussian with mean zero and variances $\sigma_{l,w}^{2}$ and $\sigma_{l,J}^{2}$, respectively. Thus, the summation of noise and jamming signal is distributed as $w_{l}\left(t\right)∼\mathrm{CN}\left(0,\sigma_{l}^{2}\right)$, where $\sigma_{l}^{2}=\sigma_{l,w}^{2}+\sigma_{l,J}^{2}$.

In contrast to Ref. [15], where scattering intensities are assumed identical, i.e., $\xi_{lk}=\xi$, l=1,2,⋯,L and k=1,2,⋯,K, in this paper we take the attenuation of each propagation channel into account, and the scattering intensities are expressed as $\xi_{lk}=\xi a_{lk}$, where $a_{lk}$ denotes the attenuation of the electromagnetic waves from the *k*th transmitter to the *l*th receiver, and usually can be calculated according to the radar equation. Then the radar echoes are rewritten as

(8) $$r_{l}\left(t\right)=\xi \sum_{k=1}^{K}a_{lk}s_{k}\left(t-\tau_{lk}\right)e^{-j2\pi f_{c}\tau_{lk}}e^{j2\pi f_{lk}t}+w_{l}\left(t\right).$$

Here, the delay $\tau_{lk}$ and Doppler shift $f_{lk}$ and the scattering coefficient ξ of the observed target are unknown parameters to estimate. With the positions of the radar transmit and receive antennas, which are known in such a distributed MIMO radar system, the location and velocity of the target are then inferred from these estimates of delay and Doppler. According to Equations (6) and (7), the estimate of the target location and velocity is also related to the geometry of radar antennas, which suggests that radar antennas contribute differently to the estimate of location and velocity.

The parameter estimation accuracy of the target is crucial for the survival of the target encountering such MIMO radars, especially when the radar operates in tracking mode. The goal of the jamming devices becomes to degrade the radar accuracy, such that the target has higher chances to escape from the track of the radar. In particular, under the constraint on the jamming power, the jammer is required to identify the contribution of each radar receiver on the radar accuracy and optimally allocate jamming resources towards these receivers at the aim of maximizing the degradation on radar accuracy.

To achieve the jamming goal, we derive the CRB of the parameter estimate in the next section, because CRB is often regarded as an evaluation of the radar accuracy. For the sake of simplifying the calculation of CRB, we further assume that the orthogonality between the radar echoes still holds when there is delay and Doppler, i.e.,

(9) $$\int_{T}s_{k}\left(t-\tau_{k}\right)s_{m}^{*}\left(t-\tau_{m}\right)e^{j2\pi (f_{k}-f_{m})}dt\approx \left\{\begin{array}{cc} 1, & k=m,\\ 0, & k\neq m. \end{array}\right.$$

## 3. Calculation of CRB

In this section, we derive the CRB of target parameters’ estimation under jamming environments. The calculated CRB is then used in the next section to construct the CRB-maximization-based jamming strategy to guide the allocation of jamming power towards receivers of the MIMO radar. Since the jamming power determines the variance of received interference, $\sigma_{l,J}^{2}$, and is related to the noise-interference variance, $\sigma_{l}^{2}=\sigma_{l,w}^{2}+\sigma_{l,J}^{2}$, we model the CRB as a function of $\sigma_{l}^{2}$. The derivation of CRB is inspired by Refs. [15,19]; however, the results in Refs. [15,19] are extended by abandoning the assumption that both $\xi_{lk}$ and $\sigma_{l}^{2}$ are identical, respectively. Please note that in the proposed jamming strategy, the interference power towards each receive antenna of MIMO can be different, which means the variances $\sigma_{l}^{2}$ are not identical.

As for the MIMO radar, the unknown parameters to estimate are

(10) $$\theta ={\left[x,y,v_{x},v_{y},R\left(\xi \right),I\left(\xi \right)\right]}^{T},$$

where R(·) and I(·) denote the real and imaginary parts of a complex-valued argument, respectively. Among these parameters only the locations and velocities are of interests, expressed as

(11) $$\rho ={\left[x,y,v_{x},v_{y}\right]}^{T}.$$

The CRB matrix of ρ is

(12) $$C_{\rho }={\left[H\left(S-V\Lambda^{-1}V^{T}\right)H^{T}\right]}^{-1},$$

where definitions of matrices S, V and Λ are given in the sequel, while we refer to Ref. [15] for the expression of H.

The diagonal elements of the CRB matrix are the CRB of the corresponding variables, respectively, i.e.,

(13) $$\left\{\begin{array}{c} CRB_{x}={\left[C_{\rho }\right]}_{1,1},\\ CRB_{y}={\left[C_{\rho }\right]}_{2,2},\\ CRB_{v_{x}}={\left[C_{\rho }\right]}_{3,3},\\ CRB_{v_{y}}={\left[C_{\rho }\right]}_{4,4}. \end{array}\right.$$

In (12), S is

(14) $$S=\left[\begin{array}{cc} S_{\tau } & S_{\tau f}\\ S_{\tau f}^{T} & S_{f} \end{array}\right],$$

where

(15) $$S_{\tau }=diag\left(\varepsilon_{11},\varepsilon_{12},\ldots ,\varepsilon_{LK}\right),$$

(16) $$S_{\tau f}=diag\left(\gamma_{11},\gamma_{12},\ldots ,\gamma_{LK}\right),$$

(17) $$S_{f}=diag\left(\eta_{11},\eta_{12},\ldots ,\eta_{LK}\right).$$

The matrix V is

(18) $$V=\left[\begin{array}{cccccccc} \mu_{11} & \mu_{12} & \ldots & \mu_{LK} & \nu_{11} & \nu_{12} & \ldots & \nu_{LK}\\ {\bar{\mu }}_{11} & {\bar{\mu }}_{12} & \ldots & {\bar{\mu }}_{LK} & {\bar{\nu }}_{11} & {\bar{\nu }}_{12} & \ldots & {\bar{\nu }}_{LK} \end{array}\right],$$

and Λ is

(19) $$\Lambda =2\sum_{l=1}^{L}\sum_{k=1}^{K}\frac{a_{lk}^{2}}{\sigma_{l}^{2}}\cdot I_{2},$$

where

(20) $$\varepsilon_{lk}=\frac{2{\left|\xi_{lk}\right|}^{2}}{\sigma_{l}^{2}}\left\{4\pi^{2}f_{c}^{2}+\int_{\tau_{lk}}^{T+\tau_{lk}}{\left|{\dot{s}}_{k}\left(t-\tau_{lk}\right)\right|}^{2}dt+2R\left[j2\pi f_{c}\int_{\tau_{lk}}^{T+\tau_{lk}}s_{k}\left(t-\tau_{lk}\right){\dot{s}}_{k}^{*}\left(t-\tau_{lk}\right)dt\right]\right\},$$

(21) $$\gamma_{lk}=\frac{2{\left|\xi_{lk}\right|}^{2}}{\sigma_{l}^{2}}R\left[-4\pi^{2}f_{c}\left({\bar{t}}_{k}+\tau_{lk}\right)+j2\pi \int_{\tau_{lk}}^{T+\tau_{lk}}t{\dot{s}}_{k}\left(t-\tau_{lk}\right)s_{k}^{*}\left(t-\tau_{lk}\right)dt\right],$$

(22) $$\eta_{lk}=\frac{2{\left|\xi_{lk}\right|}^{2}}{\sigma_{l}^{2}}R\left[\xi_{lk}j2\pi f_{c}+\xi_{lk}\int_{\tau_{lk}}^{T+\tau_{lk}}{\dot{s}}_{k}\left(t-\tau_{lk}\right)s_{k}^{*}\left(t-\tau_{lk}\right)dt\right].$$

(23) $$\mu_{lk}=-\frac{2}{\sigma_{l}^{2}}R\left[\xi_{lk}j2\pi f_{c}+\xi_{lk}\int_{\tau_{lk}}^{T+\tau_{lk}}{\dot{s}}_{k}\left(t-\tau_{lk}\right)s_{k}^{*}\left(t-\tau_{lk}\right)dt\right],$$

(24) $${\bar{\mu }}_{lk}=\frac{2}{\sigma_{l}^{2}}R\left[-\xi_{lk}2\pi f_{c}+\xi_{lk}j\int_{\tau_{lk}}^{T+\tau_{lk}}{\dot{s}}_{k}\left(t-\tau_{lk}\right)s_{k}^{*}\left(t-\tau_{lk}\right)dt\right],$$

(25) $$\nu_{lk}=-\frac{2I\left(\xi_{lk}\right)}{\sigma_{l}^{2}}2\pi \left({\bar{t}}_{k}+\tau_{lk}\right),$$

(26) $${\bar{\nu }}_{lk}=\frac{2R\left(\xi_{lk}\right)}{\sigma_{l}^{2}}2\pi \left({\bar{t}}_{k}+\tau_{lk}\right),$$

with definitions

(27) $${\dot{s}}_{k}\left(t-\tau_{lk}\right)=\frac{\partial s_{k}}{\partial (t-\tau_{lk})},$$

(28) $${\bar{t}}_{k}=\int_{T}t\left|s_{k}\left(t\right)\right|dt,$$

and $I_{n}$ denotes the *n* dimensional identity matrix.

Substituting the expressions of the baseband waveform Equation (4) into Equations (20)–(26) yields

(29) $$\varepsilon_{lk}=\frac{{\left|\xi_{lk}\right|}^{2}}{\sigma_{l}^{2}}\cdot 8\left[\pi^{2}{\left(f_{c}+k\Delta f\right)}^{2}+\frac{1}{T^{2}}\right],$$

(30) $$\gamma_{lk}=-\frac{{\left|\xi_{lk}\right|}^{2}}{\sigma_{l}^{2}}\cdot 8\pi^{2}\left(T/2+\tau_{lk}\right)\left(f_{c}+k\Delta f\right),$$

(31) $$\eta_{lk}=\frac{{\left|\xi_{lk}\right|}^{2}}{\sigma_{l}^{2}}\cdot 8\pi^{2}\cdot \frac{{\left(T+\tau_{lk}\right)}^{3}-\tau_{lk}^{3}}{3T},$$

(32) $$\mu_{lk}=\frac{I\left(\xi_{lk}\right)}{\sigma_{l}^{2}}\cdot 4\pi \left(f_{c}+k\Delta f\right),$$

(33) $${\bar{\mu }}_{lk}=-\frac{R\left(\xi_{lk}\right)}{\sigma_{l}^{2}}\cdot 4\pi \left(f_{c}+k\Delta f\right),$$

(34) $$\nu_{lk}=-\frac{I\left(\xi_{lk}\right)}{\sigma_{l}^{2}}\cdot 4\pi \left(T/2+\tau_{lk}\right),$$

(35) $${\bar{\nu }}_{lk}=\frac{R\left(\xi_{lk}\right)}{\sigma_{l}^{2}}\cdot 4\pi \left(T/2+\tau_{lk}\right).$$

With the CRB versus the power distributed for the *l*th receive antenna, $\sigma_{l}$, it is possible to construct the CRB maximization problem, as discussed in next section. By optimizing $\sigma_{l}$, the jammer possibly degrades the accuracy of the MIMO radar such that the target is better protected.

## 4. Barrage Jamming Strategy Design

### 4.1. Optimization Model

With the calculated CRB, we develop a barrage jamming strategy against the distributed MIMO radar. We first cast the strategy design as an optimization problem, aiming at maximizing the CRB, with the power allocated to receive antennas of MIMO radar as the variables, under the constraint of fixed power budget for interference. Then a generic optimization algorithm is used to solve the maximization problem, and the complexity issues of the algorithm are briefly discussed.

Before introducing the jamming model, we make the following assumptions on the distributed MIMO radar and the jammer. Since most of the existing radars have complete transmit and receive units, it is assumed that the MIMO radar consists of multiple nodes with independent and complete transmit and receive functions. In addition, the nodes are exactly synchronized and fully coherent with each other. In this case, the numbers of the receive and transmit antennas are the same, i.e., K=L, as well as the positions of the corresponding antennas, i.e., $\left(x_{k}^{t},y_{k}^{t}\right)=\left(x_{l}^{r},y_{l}^{r}\right)$. We consider the scenario of on-board self-defense jamming, where the target (of the radar) is collocated with the jammer. By transmitting noise-like signals to MIMO radar’s receive antennas, the jammer aims at reducing the radar accuracy, hence protects the target from being tracked or locked by the radar [20,21]. With the application of active phased array technology [22], it is possible for the jammer to simultaneously transmit barrage jamming signals in diverse directions. We assume that the jammer acquires some basic information of the radar [23], e.g., locations of the nodes, through on-board reconnaissance technology or pre-loaded messages from other intelligence agencies, such that calculation of the CRB is possible.

**Figure 2:**
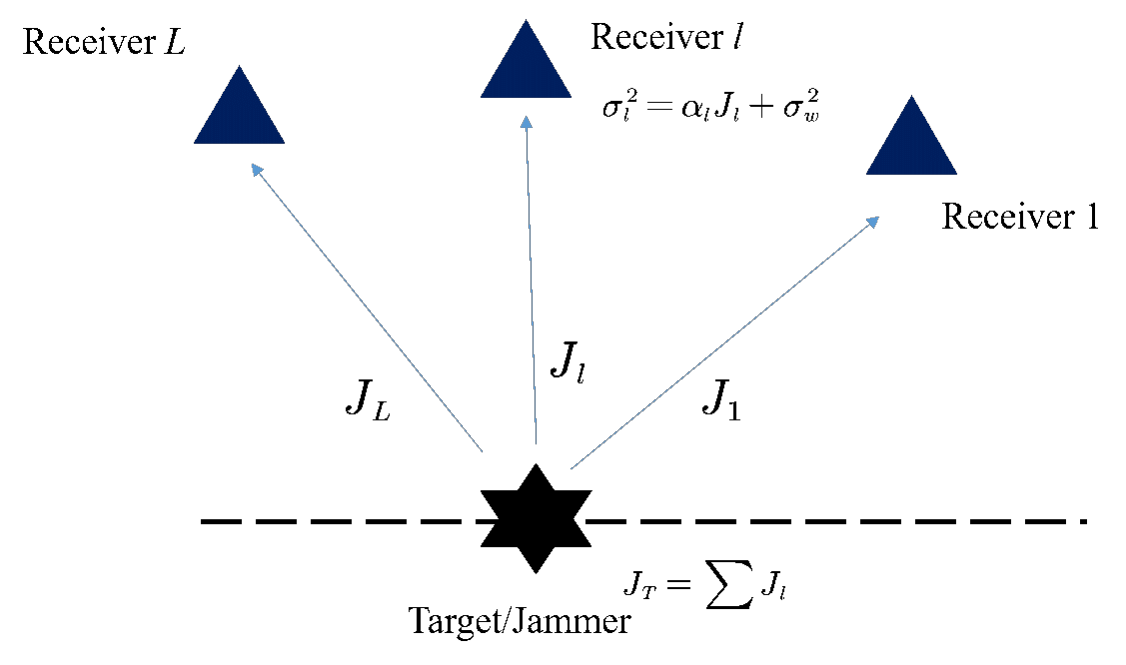
Power allocation of the jammer.

The power allocation of the jammer is illustrated in Figure 2. Denote by $J_{1},J_{2},\ldots ,J_{L}$ the transmitting power towards the 1st, 2nd, *…*, and the *L*th receive antenna of radar, respectively. After attenuation, the power of jamming signal received by the *l*th receive channel is

(36) $$\sigma_{l,J}^{2}=\alpha_{l}J_{l},$$

where $\alpha_{l}$ is the attenuation factor of the *l*th receive channel, and is inversely proportional to the square of the distance from the jammer to the *l*th receive antenna. Assume that the receiver noise variance of each receive antenna is identical, i.e., $\sigma_{w}^{2}=\sigma_{l,w}^{2}$, l=1,⋯,L. The variance of the additive jamming signal and noise received by the *l*th receiver is

(37) $$\sigma_{l}^{2}=\alpha_{l}J_{l}+\sigma_{w}^{2}.$$

For the jammer, the total power of the transmission is constrained as $J_{T}$, i.e.,

(38) $$J_{1}+J_{2}+\ldots +J_{L}=J_{T}.$$

Under the energy budget constraint, we need to optimize the power distribution, $J_{1},J_{2},\ldots ,J_{L}$, to affect the radar accuracy as much as possible.

We then construct the optimization problem for power allocation by maximizing the CRB. Recall that the CRB of target parameters can be expressed as functions of receiver interference-and-noise variances, $\sigma_{l}^{2}$, i.e.,

(39) $$\begin{array}{c} f_{x}\overset{\Delta }{\;=}CRB_{x}\left(\sigma_{1}^{2},\sigma_{2}^{2},\cdots ,\sigma_{L}^{2}\right),\\ f_{y}\overset{\Delta }{\;=}CRB_{y}\left(\sigma_{1}^{2},\sigma_{2}^{2},\cdots ,\sigma_{L}^{2}\right),\\ f_{v_{x}}\overset{\Delta }{\;=}CRB_{v_{x}}\left(\sigma_{1}^{2},\sigma_{2}^{2},\cdots ,\sigma_{L}^{2}\right),\\ f_{v_{y}}\overset{\Delta }{\;=}CRB_{v_{y}}\left(\sigma_{1}^{2},\sigma_{2}^{2},\cdots ,\sigma_{L}^{2}\right), \end{array}$$

which correspond to the position and velocity parameters of the target along with *x* and *y* axes, respectively. Maximizing the weighted average of aforementioned CRB yields

(40) $$\begin{array}{c} \max_{J_{1},J_{2},\ldots ,J_{L}}\lambda_{x}f_{x}+\lambda_{y}f_{y}+\lambda_{v_{x}}f_{v_{x}}+\lambda_{v_{y}}f_{v_{y}},\\ \begin{array}{cc} s.t. & J_{1}+J_{2}+\ldots +J_{L}=J_{T},\\ & \sigma_{l}^{2}=\alpha_{l}J_{l}+\sigma_{w}^{2}, \end{array} \end{array}$$

where $\lambda_{x}$, $\lambda_{y}$, $\lambda_{v_{x}}$, $\lambda_{v_{y}}$ are regularization factors that represent the importance of the corresponding parameters, respectively.

### 4.2. Optimization Solver

Since the CRB expressions are very complicated and highly non-convex, we apply a heuristic algorithm to solve this optimization problem. Popular modern heuristic algorithms include genetic algorithm, simulated annealing, tabu search, ant colony optimization, etc. [24]. In particular, we choose particle swarm optimization (PSO) [25] as the solver because it is very simple and easy to implement [24].

To implement the PSO algorithm, we apply the particleswarm function in MATLAB software and use default parameters. PSO was first proposed in 1995, and is inspired from swarm behavior such as bird flocking and schooling in nature [24]. PSO is initialized by randomly generating some “particles” within the feasible region of the problem. During iterative motions of particles, every particle remembers its own best location and knows the global optimal position of all the particles, as well as the corresponding values of the objective function. The next movement of the particle is determined by the distances from the current position to its own best and the global optimal position, and some random coefficients. A more detailed discussion of the procedure of PSO and parameter selection can be found in Refs. [26,27].

Solving the optimization problem Equation (40) with PSO algorithm, we obtain the power allocation for each radar receiver, i.e., $J_{1},J_{2},\ldots ,J_{L}$, which affects the accuracy of the MIMO radar. In the next section, numerical results are carried out to examine the performance of the proposed jamming strategy.

### 4.3. Complexity Issues

The computational burden depends on the complexity of calculating the cost function, the number of particles in each iteration and the number of iterations to achieve convergence.

When we calculate the CRB matrix via Equation (12), matrix multiplication and inverse operations are involved. Please note that the matrices H, S, V and Λ are of dimension 4×2LK, 2LK×2LK, 2LK×2 and 2×2, respectively. Thus, we find that the number of multiplication operations is in the scale of $O\left(L^{2}K^{2}\right)$.

Due to the nonlinearity of the cost function and the stochastic manner of the PSO algorithm, analytic prediction of the number of iterations is rather difficult. Generally, as the dimension of the problem, *L*, increases, PSO algorithm suffers from performance deteriorates as the search space increases [28]. To complete the calculation in a predictable time, one can fix the maximum number of particles and the maximum number they iterate according to the computational capacity of the processor in practice.

There may be several methods to shorten the solution time and implement the algorithm in real conditions; however, we leave this for future investigation. For example, Tsiropoulou et al. in Ref. [29] consider a game problem towards determining the equilibrium powers for each passive Radio Frequency Identification (RFID) tag, where each tag has its own cost function, and solve the problem in a distributed manner to reduce computational burden. Similar idea can also be found in Ref. [30], which exploits the separability of the cost functions and optimizes each dimension independently to avoid the “curse of dimensionality” of the PSO algorithm. However, due to the complexity of the CRB matrix (12), it is hard to obtain the analytic expressions of the cost functions (39), and it is still an open question how to separate these cost functions (39) with respect to the jamming resource towards each radar receive antenna or define a proper utility function for each receiver as in Ref. [29]. A greedy approach to PSO is developed in Ref. [28], which uses one-dimensional swarms, then searches these swarms separately, and integrates the searches together by a global swarm [30]. Furthermore, one can also apply parallel PSO in a cluster with multiple nodes [31] or a Graphic Processing Unit (GPU) [32].

## 5. Numerical Experiments

We perform numerical experiments to compare the effectiveness of different jamming power allocation methods, including (1) uniform (the power is uniformly allocated towards all the receive antennas of the MIMO radar) (2) concentrated (all the jamming power is concentrated on a certain receiver), (3) random (the jamming power is randomly distributed over radar receivers), and (4) optimized (the proposed allocation method). The CRB of the position or velocity of the target is examined as the metric to evaluate the effects of different allocation methods on the distributed MIMO radar. The carrier frequency of the radar is $f_{c}=10$ GHz, the interval between each frequency channel is Δf=100 MHz, and the coherent processing interval is T=1 s. Two target-radar scenarios are considered, as discussed in the sequel. In both cases, the target position is set as the origin point, i.e., (x,y)=(0,0).

### 5.1. First Target-Radar Scenario

In the first scenario, the velocity of the target is $v_{x}=100$ m/s, $v_{y}=300$ m/s, and the MIMO radar contains three nodes (denoted by “Node 1”, “Node 2”, “Node 3”, respectively) of which the transmit and receive antennas are located at $\left(x_{1}^{t},y_{1}^{t}\right)=\left(x_{1}^{r},y_{1}^{r}\right)=\left(15,1\right)$ km, $\left(x_{2}^{t},y_{2}^{t}\right)=\left(x_{2}^{r},y_{2}^{r}\right)=\left(1,2\right)$ km, $\left(x_{3}^{t},y_{3}^{t}\right)=\left(x_{3}^{r},y_{3}^{r}\right)=\left(1,1\right)$ km, respectively. In the concentrated allocation, the power is concentrated on the first receive antenna at $\left(x_{1}^{r},y_{1}^{r}\right)$. Without loss of generality, we set the CRB of $v_{x}$, $f_{v_{x}}$, as the cost function, i.e., $\lambda_{v_{x}}=1$ and $\lambda_{x}=\lambda_{y}=\lambda_{v_{y}}=0$ in Equation (40).

**Figure 3:**
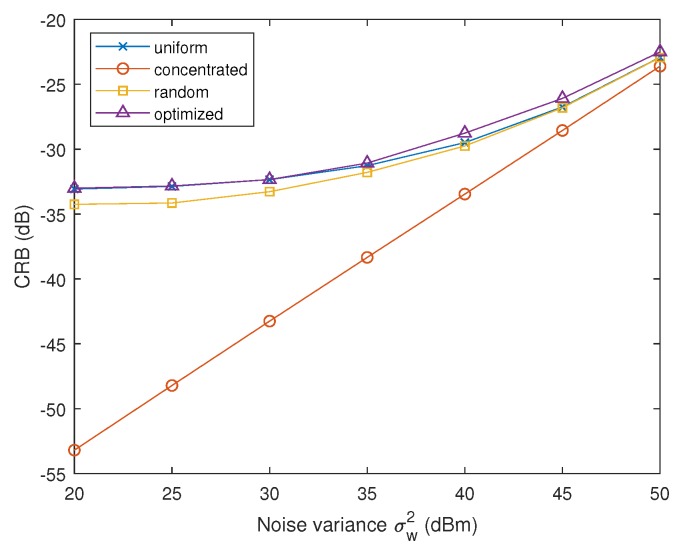
CRB of $v_{x}$ versus the noise variance of radar receiver in the first experiment of Scenario 1.

We first evaluate the impact of noise variance $\sigma_{w}^{2}$ of radars’ receivers. The noise variance is varied from 20 dBmW to 50 dBmW, while the total jamming power is $J_{T}=50$ dBm. The allocation strategy obtained by PSO is shown in Table 1, and CRB versus noise variance of the radar receiver is shown in Figure 3.

Please note that the distances between the three radar nodes and the target are different: Node 1 is the furthest and Node 3 is the nearest one. From Table 1, we find that the optimized jamming strategy tends to concentrate the power towards the nearest radar node, i.e., Node 3. As the noise variance of the radar receiver increases, the concentration of the jamming power becomes more distinct. Because in low signal-to-noise ratio (SNR) schemes, the nearest radar node contributes most to the overall estimation accuracy of the MIMO radar due to its highest SNR. As the noise variance becomes significant, the contribution (in the sense of MIMO radar accuracy) of the far located nodes is negligible, hence the jamming power is no longer necessary for these radar nodes.

Figure 3 shows that the proposed strategy leads to maximum CRB, which suggests that the MIMO radar have the worst estimation accuracy of the $v_{x}$ parameter. Centralizing the interference energy towards radar Node 1 results in the lowest CRB, because Node 1 is the most distant node enjoying the lowest SNR and thus contributing least to the radar accuracy.

**Figure 4:**
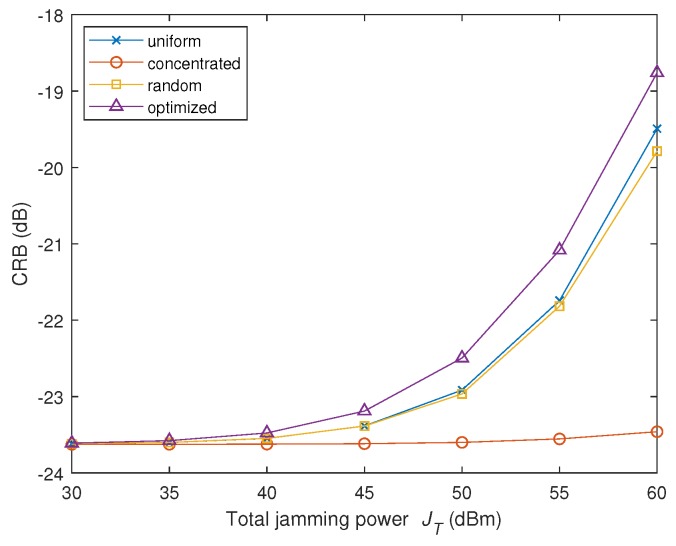
CRB of $v_{x}$ versus the total jamming power $J_{T}$ in the second experiment of Scenario 1.

In the second experiment, the impact of the total jamming power $J_{T}$ on the CRB is tested. The power budget $J_{T}$ is changed from 30 dBmW to 60 dBmW, and the noise variance $\sigma_{w}^{2}=50$ dBmW is fixed. Under this condition, the energy towards radar nodes provided by the proposed strategy is shown in Table 2, and the CRB versus total jamming power $J_{T}$ is shown in Figure 4.

We observe from Table 2 the phenomenon similar to that in the first experiment, which is the optimized jamming strategy tends to concentrate the power towards the nearest radar node, i.e., Node 3, when the jamming power is less. Along with the increase of $J_{T}$, more jamming power is allocated against the Node 2. As aforementioned, the three radar nodes do not contribute equally to the radar accuracy, because the distance to the target is different. In this low SNR scheme ($\sigma_{w}^{2}=50$ dBmW), the nearest radar node (i.e., Node 3) contributes most to the overall estimation accuracy of the MIMO radar. When the interference power budget is less ($J_{T}\leq 50$ dBmW), the energy is centered at Node 3. As the power budget is increased, the importance of Node 2 is manifested, and some jamming energy is spared towards Node 2.

Figure 4 also demonstrates that the proposed strategy enjoys the optimum performance in comparison with other methods, while the concentrated method ranks the worst. The reason is that Node 1 is located furthest and contributes least to the overall accuracy of the MIMO radar accuracy. Destroying Node 1 (by concentrating all the power on it) does not affect much on the accuracy of the MIMO radar.

### 5.2. Second Target-Radar Scenario

**Figure 5:**
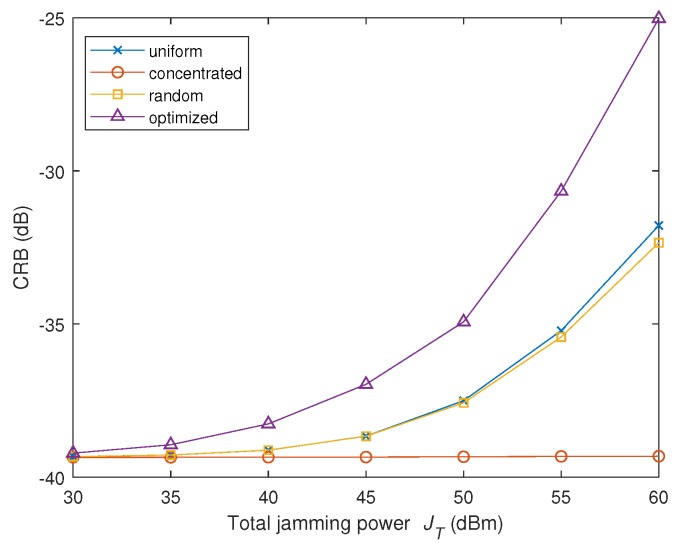
CRB of $v_{x}$ versus the total jamming power $J_{T}$ in the second scenario.

**Figure 6:**
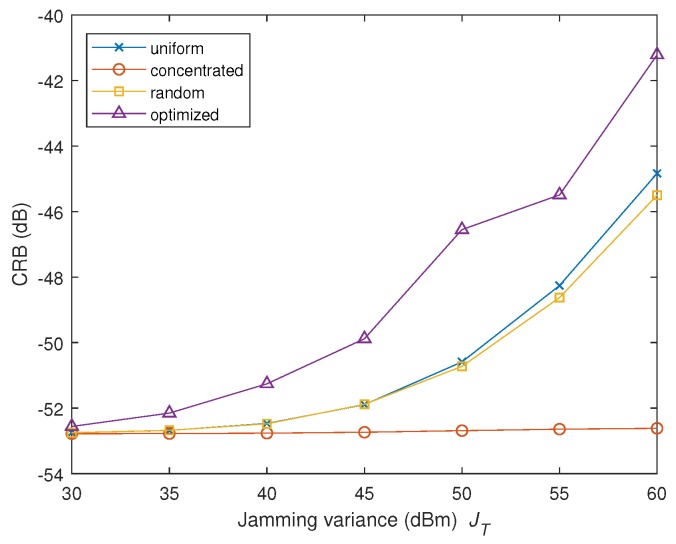
CRB of *x* versus the total jamming power $J_{T}$ in the second scenario.

In the second scenario, we increase the number of radar nodes to K=L=8, whose positions are random and satisfy that $0\leq x_{k}^{t}=x_{k}^{r}\leq 10$ km, $0\leq y_{k}^{t}=y_{k}^{r}\leq 10$ km. The total interference power $J_{T}$ varies from 30 dBm to 60 dBm, and the noise power of the receiver is 40 dBm. Other parameters are consistent with the previous experiment. We consider the CRB of the target speed parameter $v_{x}$ and the location parameter *x*. The results are depicted in Figure 5 and Figure 6, respectively.

Curves in both Figure 5 and Figure 6 demonstrate the superiority of the proposed strategy (leading to highest CRB) among all the algorithms under test. Compared to Figure 4 and Figure 5, we find that the gap between the proposed strategy and the rest becomes larger in comparison with the results in the first scenario. It can be concluded that in this more complicated scenario, the advantage of the proposed algorithm over the traditional methods becomes more significant.

To summarize, according to the experiment results in both target scenarios, we find that the proposed algorithm achieves better performance than the rest traditional methods. In the uniform, concentrated, and random allocation of jamming power, the geometry of MIMO antennas is not considered, and the jammer fails to make full use of the jamming power. In our proposed method, the geometry is used and the CRB function is maximized, which can be regarded as a quantified evaluation on the importance of all radar receivers, so as to efficiently allocate the jamming power to those receivers that are more important to the radar accuracy.

## 6. Conclusions

We derive the CRB of the target motion and location parameters estimation when using distributed MIMO radar under jamming environments, and propose a jamming strategy towards the MIMO radar based on the CRB. The strategy is modeled as optimization of jamming power allocation, with the cost function maximizing the CRB of the motion and location parameters, under the constraint that the total jamming power is constant. Due to the non-convexity of the optimization problem, PSO is used to solve the problem. Numerical experiments are executed to evaluate the jamming strategy in comparison with some traditional strategies. The results demonstrate that the proposed strategy outperforms the traditional ones.

Since the PSO is usually regarded as having high computational complexity, future investigation on accelerating the algorithm would contribute to implementing the method in practical equipment, especially in those time-critical applications.

## Figures and Tables

**Table 1:** Optimized interference power towards 3 radar nodes versus noise variances in the first experiment of Scenario 1.

Noise Variance $\sigma_{w}^{2}$ (dBm)	Towards Node 1 (W)	Towards Node 2 (W)	Towards Node 3 (W)
20	39.28	30.74	29.98
25	37.65	31.48	30.87
30	32.48	33.83	33.68
35	16.15	41.27	42.58
40	0	43.85	56.15
45	0	29.86	70.14
50	0	0	100

**Table 2:** Optimized interference power towards 3 radar nodes versus total jamming power in the second experiment of Scenario 1.

Total Jamming Power $J_{T}$ (dBm)	Towards Node 1 (W)	Towards Node 2 (W)	Towards Node 3 (W)
30	0	0	1
35	0	0	3.16
40	0	0	10
45	0	0	31.16
50	0	0	100
55	0	94.42	221.80
60	0	438.46	561.54

## Abbreviations

The following abbreviations are used in this manuscript:

CRB	Cramer–Rao bound
ECCM	electronic counter-counter measures
GPU	Graphic Processing Unit
MIMO	multiple input multiple output
PSO	particle swarm optimization
RFID	Radio Frequency Identification
SNR	signal-to-noise ratio
